# Interactive Effects of Seminal Plasma and *Sonchus oleraceus* L. Aqueous Extract on Post-Thaw Sperm Kinematics, Oxidative Status, and In Vitro Fertilization Outcomes in Ram Semen

**DOI:** 10.3390/ani16193042

**Published:** 2026-09-27

**Authors:** Hela Derbali, Wijden Niama, Mohammedreza Ebrahimi, Sihem Amiri, Fabrizio Chessa, Ignazio Cossu, Valeria Pasciu, Francesca Daniela Sotgiu, Fiammetta Berlinguer, Maria Dattena, Mokhtar Mahouachi, Samia Ben Saïd, Laura Mara

**Affiliations:** 1 Laboratoire d’Appui à la Durabilité des Systèmes de Production Agricole dans la Région du Nord-Ouest, École Supérieure d’Agriculture du Kef (ESAK), Université de Jendouba, Jendouba 8189, Tunisia; derbalihela20@gmail.com (H.D.); naaymawejden@gmail.com (W.N.); taymallahmah@gmail.com (M.M.); 2 School of Biosciences, University of Nottingham, Sutton Bonington Campus, College Road, Leicestershire LE12 5RD, UK; mohammadreza.ebrahimi@nottingham.ac.uk; 3 Department of Animal Science, Agricultural Research Agency of Sardinia, 07100 Sassari, Italy; fchessa@agrisricerca.it (F.C.); icossu@agrisricerca.it (I.C.); mariadattena@gmail.com (M.D.); 4 Department of Veterinary Medicine, University of Sassari, Via Vienna 2, 07100 Sassari, Italy; vpasciu@uniss.it (V.P.); fdsotgiu@uniss.it (F.D.S.); berling@uniss.it (F.B.); 5 Ecole Supérieure d’Agriculture de Mateur, University of Carthage, Carthage 1054, Tunisia; sihem.amiriii@gmail.com

**Keywords:** ram semen, cryopreservation, seminal plasma, *Sonchus oleraceus*, oxidative stress, in vitro fertilization

## Abstract

This study investigated the combined effects of seminal plasma (SP) and *Sonchus oleraceus* L. aqueous extract on post-thaw sperm quality and fertilizing ability in rams. Semen collected from seven adult rams was pooled and divided into six experimental groups according to the presence (SP+) or absence (SP−) of SP and supplementation with 0, 0.5, or 2 g/L of *Sonchus oleraceus* extract. Samples were cryopreserved in a Tris–egg yolk–citric acid extender. Post-thaw sperm quality using computer-assisted sperm analysis (CASA), hypo-osmotic swelling (HOS) test, and oxidative stress biomarkers, including reactive oxygen species (ROS), malondialdehyde (MDA) and total thiols as antioxidative parameter were evaluated. Removal of SP significantly enhanced several kinematic parameters (VCL, ALH, DCL) and reduced abnormal morphology (*p* < 0.05), whereas its presence improved motion quality indicators such as beat cross frequency (BCF), linearity (LIN), and straightness (STR) (*p* < 0.05). However, SP removal induced a marked oxidative imbalance, characterized by increased ROS and MDA levels and decreased total thiols (*p* < 0.001). Supplementation with *S. oleraceus* extract improved membrane integrity and restored redox balance by increasing thiols levels (*p* = 0.0001) and reducing lipid peroxidation (*p* < 0.001), with significant SP × dose interactions. Based on these results, in vitro fertilization (IVF) was performed only using SP+ groups, as the presence of SP ensured a more physiologically relevant environment and maintained oxidative balance necessary for fertilization. No significant differences were detected in cleavage or blastocyst rates among the three *S. oleraceus* extract doses. These findings highlight an interaction between SP and *Sonchus oleraceus* aqueous extract, suggesting that combining endogenous protein-rich components with exogenous plant-derived antioxidants may enhance post-thaw sperm functionality and its oxidative status in rams.

## Simple Summary

Cryopreservation of ram semen is an important tool in animal reproduction; however, spermatozoa are susceptible to cryodamage during the freezing–thawing process, which may compromise their functional quality and fertilizing capacity. This study investigated whether removing seminal plasma and adding a plant extract from *Sonchus oleraceus* could improve the quality of cryopreserved ram semen. Semen samples were divided into different groups depending on the presence or absence of seminal plasma and different doses of plant extract, then frozen and later thawed for analysis. Sperm quality was evaluated using computer-assisted analysis, membrane integrity tests, and measurements of oxidative stress indicators, as well as in vitro fertilization performance. The results showed that removing seminal plasma improved some movement characteristics of sperm but also increased cellular damage caused by oxidative stress. In contrast, supplementation with *Sonchus oleraceus* extract reduced this damage, improved sperm membrane integrity, and restored the balance between harmful and protective molecules. However, *Sonchus oleraceus* aqueous extract supplementation did not affect in vitro embryo development. Overall, this study suggests that plant-based antioxidants may help improve ram semen quality during cryopreservation and support more efficient assisted reproduction techniques in sheep breeding.

## 1. Introduction

Semen cryopreservation is a key technology in sheep reproduction, supporting genetic preservation, artificial insemination, disease control and the dissemination of superior genetic traits [1,2]. However, ram semen remains particularly sensitive to cryopreservation compared to other domestic species [3]. The high proportion of polyunsaturated fatty acids within the sperm plasma membrane is considered as the major factor rendering spermatozoa particularly vulnerable to ROS-mediated oxidative [4,5]. Furthermore, freeze–thaw processes expose sperm cells to thermal shock, osmotic imbalance, and intracellular ice crystal formation, leading to impaired motility, membrane damage, and reduced fertilizing capacity [6,7,8,9]. To overcome these limitations, considerable efforts have focused on improving semen extenders and incorporating antioxidant strategies to preserve sperm structure and function during cryopreservation. Among these approaches, two main strategies have emerged. The first involves the use of seminal plasma (SP), a complex biological fluid containing proteins, enzymes, sugars, and antioxidants [10,11]. SP has been shown to stabilize sperm membranes, reduce cold shock, and modulate oxidative stress during cryopreservation [12,13,14]. However, its role remains controversial. While several studies report beneficial effects on post-thaw sperm quality and fertilizing ability [8,11], others have shown detrimental or inconsistent effects depending on its composition and handling conditions [15,16,17]. This dual effect suggests that SP may exert both protective and deleterious actions during cryopreservation.

The second strategy focuses on the use of exogenous antioxidants, particularly plant-derived compounds. Plant extracts are rich sources of polyphenols, flavonoids, and other bioactive molecules capable of scavenging ROS, reducing lipid peroxidation, and enhancing sperm resistance to cryo-induced stress [18,19]. Several studies in rams have demonstrated that plant extracts can improve membrane integrity, reduce DNA damage, and enhance fertilization outcomes following semen preservation [20,21,22].

Among these natural resources, *Sonchus oleraceus* L. represents a promising candidate due to its rich phytochemical composition, including polyphenols, flavonoids, and vitamins with strong antioxidant properties [23]. Its ability to scavenge free radicals and mitigate oxidative stress has been demonstrated in previous studies [24,25]. However, despite its antioxidant potential, the application of *Sonchus oleraceus* aqueous extract in semen cryopreservation has not been investigated, and its interaction with SP during sperm cryopreservation remains unknown.

Thus, considering the complementary nature of SP (a source of proteins and endogenous protective factors) and plant extracts (a source of exogenous antioxidants), their combined use may provide complementary protection against cryodamage. Accordingly, the present study investigated the effects of SP, both individually and in combination with *Sonchus oleraceus* aqueous extract on cryopreservation-induced sperm damage in rams, with particular emphasis on post-thaw sperm kinematics, membrane integrity, oxidative stress status, and in vitro fertilization outcomes.

## 2. Materials and Methods

### 2.1. Ethical Issues

This study was conducted in accordance with the approval of the Ethical Local Committee (No. prot. 45461), complied with the applicable regulations including Italian Legislative Decree n. 26/2014, implementation/application of the Directive of European Union n. 2010/63/EU on the protection of animals useful for scientific purposes; Article 2, point F. The use of seminal material was as authorized under Ministerial Decree Number 02/2018-UT issued on 13 March 2018.

### 2.2. Plant Material

#### 2.2.1. Plant Collection and Aqueous Extract Preparation

*Sonchus oleraceus* L. (Asteraceae) was botanically identified based on its diagnostic morphological characteristics, including deeply lobed, glabrous leaves with acute and spreading auricles, yellow ligulate flowers arranged in capitula, and brownish achenes bearing a white pappus; the species was originally described by Carl Linnaeus (L.) [26]. The historical type material (lectotype) is preserved in the Linnaean Herbarium (LINN), managed by the Linnean Society of London, under voucher number LINN 946.9, with the lectotype designation attributed to Loutfy Boulos (1973) [26]. Whole plants of *Sonchus oleraceus* L. (stems, roots, leaves and flowers) were collected at the flowering stage from the Kef, northwest of Tunisia (36.17° N, 8.70° E) on April 2023. After collection, plant material was air-dried at room temperature and subsequently ground into a fine powder using a grinder (SK 100 standard, Retsch, Haan-Gruiten, Germany). The resulting powder was kept in a closed container at room temperature until further processing.

For aqueous extraction, 10 g of dried plant powder was suspended in 1 L of distilled water and maintained at room temperature under continuous stirring for 4 h. The resulting mixture was first filtered through Whatman No. 1 filter paper, followed by sterile filtration using 0.22 µm syringe filters (Millex^®^,Merck, Darmstadt, Germany). The resulting aqueous extract was divided into aliquots and stored at −20 °C until use. The extraction yield of the plant was determined as described by [27]. Briefly, 5 mL of the aqueous extract was transferred into a pre-weighed Petri dish and dried at 35 °C until constant weight. The dry extract mass was calculated from the difference between the final and tare weights, the 5 mL aliquot was then extrapolated to the total extract volume, and the extraction yield was calculated relative to the initial plant powder weight, as follows: Yield of extract (%) = (Weight of the obtained dry extract (g)/Weight of the plant powder used (g)) × 100 [27].

#### 2.2.2. Total Polyphenol Content Assessment

The total polyphenol content of the aqueous extracts obtained following 4 h of maceration was quantified using the Folin–Ciocalteu assay according to the procedure described by Singleton and Rossi [28]. Absorbance was measured at 765 nm using a UV–Vis spectrophotometer (Genesys 10 UV, Thermo Fisher Scientific, Monza, Italy). Gallic acid was used to generate the calibration curve over a concentration range of 0–200 µg/mL. Phenolic content was expressed as milligrams of gallic acid equivalents per gram of dry weight (mg GAE/100 mL).

### 2.3. Animal Management, Semen Collection and Assessment

Seven adult Sardinian rams (2–4 years old) enrolled in the breed genetic improvement programs by the Associazione Nazionale della Pastorizia (ASSO.NA.PA.) were used in this study. Animals were housed at the Genetic Centre of the research institute Agris Sardegna (Bonassai, Olmedo, Italy) under uniform sanitary, nutritional, and environmental conditions, with ad libitum access to water, and hay, supplemented with concentrate.

Semen was collected twice weekly for five weeks using an artificial vagina pre-warmed to 38 °C. Immediately after collection, ejaculates were evaluated macroscopically for volume and color, then they maintained in a water bath (38 °C) until further analysis. Sperm motility and morphology parameters were analyzed using a Computer-Assisted Sperm Analysis (CASA; Ceros II, Hamilton-Thorne, Beverly, MA, USA). Briefly, 10 µL of semen, regardless of the initial sperm concentration, was diluted in 1 mL of physiological saline solution (0.9% NaCl; 308 mOsm/L; pH: 4.5–7.0), then evaluated under a phase-contrast microscope using pre-warmed slide (Leja slides, 20 µm, IMV Technologies, L’Aigle, France) maintained at 38.5 °C. Five microscopic fields were evaluated in triplicate. Sperm concentration, total motility, progressive motility and normal morphology were evaluated automatically by the CASA system. A total of 70 ejaculates fulfilled the predefined quality criteria and were included in the pooling procedure. The exact number of independent collection-day pools used for cryopreservation and subsequent analyses was 10. Only ejaculates meeting the following minimum quality criteria were selected for further use: sperm concentration ≥ 3 × 10^9^ spermatozoa/mL, total motility > 60%, progressive motility > 30% and normal morphology > 70% [29,30].

### 2.4. Semen Extender Preparation

The base extender formulation was prepared according to protocols commonly used for ram semen preservation [31,32] and divided into three aliquots. The *Sonchus oleraceus* aqueous extract, previously diluted in distilled water, was added to each aliquot to obtain final concentrations of 0 g/L (control), 0.5 g/L and 2 g/L of plant powder equivalent/L. Each formulation was homogenized after being added to the base extender. Subsequently, glycerol and egg yolk were added to final concentration of 5% and 15%, respectively. The prepared extenders were kept at 37 °C in a water bath until use.

### 2.5. Semen Dilution and Freezing

At each collection day and following the initial semen evaluation, ejaculates were pooled to minimize individual variability, homogenized, and divided into six aliquots. Three aliquots were centrifuged (10 min, 600× *g*) at room temperature [33] to remove SP, generating samples without SP (SP−). After centrifugation, the seminal plasma supernatant was carefully removed without disturbing the sperm pellet. The remaining three aliquots were directly used (without centrifugation) and served as samples with seminal plasma (SP+).

Each aliquot, either SP+ or SP−, was then diluted with the corresponding extender containing the plant extract at final concentrations of 0 g/L (control), 0.5 g/L, and 2 g/L to reach a final sperm concentration of 320 × 10^6^ spermatozoa/mL [34]. After dilution, sperm motility and morphology were evaluated. The diluted samples were then cooled by placing them in a beaker containing water at 30 °C alongside a 20 mL glass ampoule of glacial acetic acid. Once the samples reached 4 °C, sperm motility and morphology were assessed again. Samples were then loaded into 0.25 mL straws (IMV Technologies, France), sealed, and equilibrated at 4 °C for 2 h in a refrigerated room.

**Figure 1:**
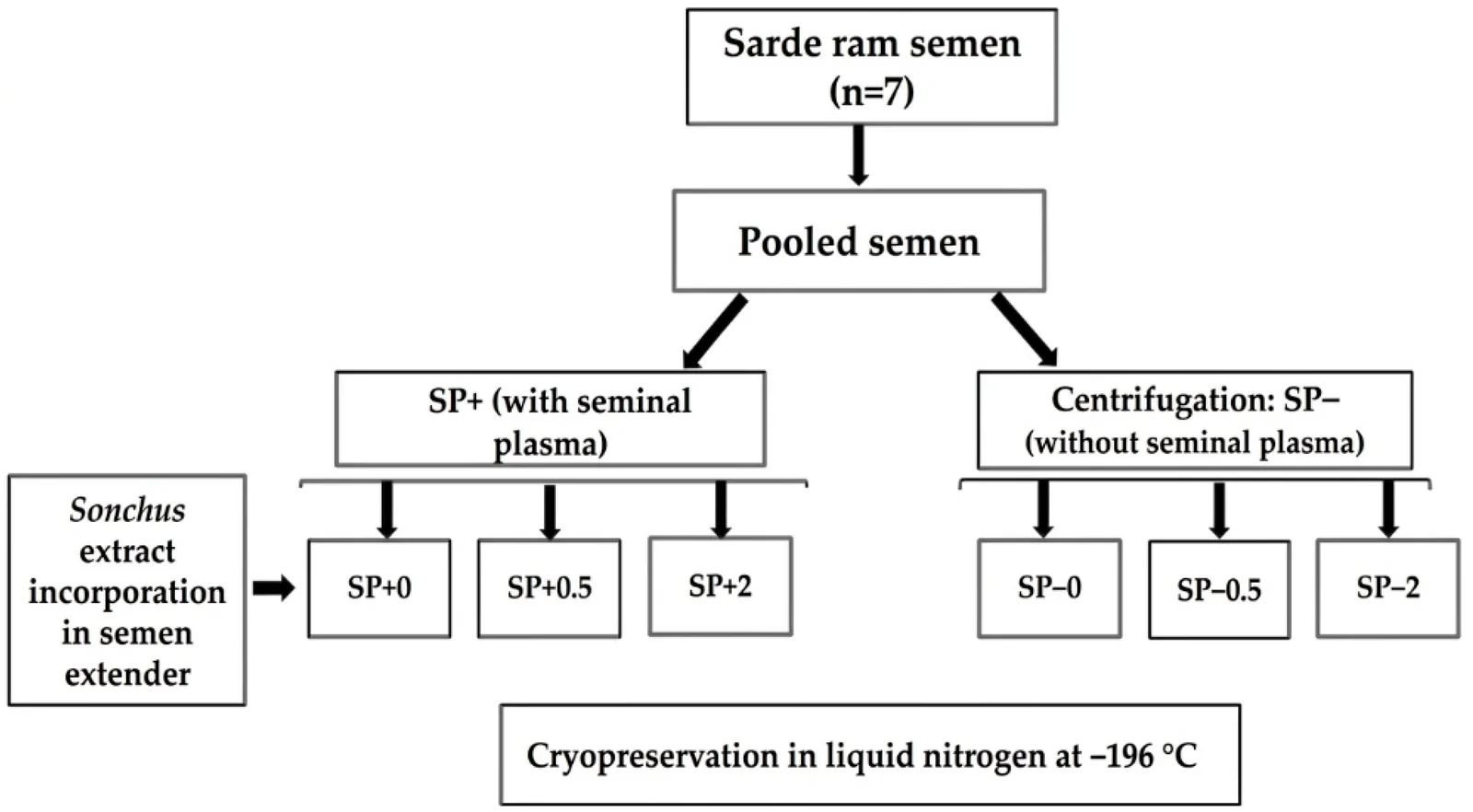
Experimental design and treatment groups in the presence or absence of seminal plasma supplemented with different concentrations of *Sonchus oleraceus* L. aqueous extract.

The straws were then frozen using an automatic freezer (Cryobath 007263, IMV Technologies, France) programmed from 5 °C to −140 °C at a cooling rate of −12.5 °C/min. Straws were then immersed into liquid nitrogen and stored until further analysis (Figure 1).

### 2.6. Post-Thaw Semen Evaluation

After four weeks, the straws were thawed by immersion in a 37 °C water bath for 30 s. The samples were evaluated immediately, including kinematics parameters, membrane integrity, and biochemical and oxidative analyses (MDA, ROS, and total thiols).

#### 2.6.1. Motility and Kinematic Parameters

After thawing, 100 µL of each diluted semen aliquot was suspended in 1 mL of physiological saline and incubated at 37 °C for 5 min. Sperm samples characteristics were subsequently evaluated using the computer-assisted sperm analysis system (CASA; Ceros II, Hamilton-Thorne Biosciences, Beverly, MA, USA). The following parameters were recorded: normal morphology (%), total motility (%), progressive motility (%), distance average path (DAP, µm), distance curved line (DCL, µm), distance straight line (DSL, µm), elongation (%), curvilinear velocity (VCL, µm/s), straight-line velocity (VSL, µm/s), average path velocity (VAP, µm/s), amplitude of lateral head displacement (ALH, µm), sperm head area (µm^2^), beat cross frequency (BCF, Hz), linearity (LIN, %), straightness (STR, %) and wobble (WOB, %).

#### 2.6.2. Plasma Membrane Integrity

Sperm membrane integrity was evaluated using the hypo-osmotic swelling test (HOST), using a hyposmotic solution (150 mOsm/L, prepared by dissolving 1.351 g of fructose and 0.735 g of sodium citrate in 100 mL distilled water). For this assay, 10 μL of sperm sample was mixed with 1000 μL of the hyposmotic solution and incubated at 37 °C for 30 min as described by [35]. After incubation, 200 spermatozoa were evaluated under a light microscope (×400). Spermatozoa showing swollen and coiled tails were considered HOST-positive (HOS+), indicating damaged spermatozoa [36].

#### 2.6.3. Oxidative Stress Status

##### Reactive Oxygen Species (ROS) Level

ROS production in cryopreserved ram semen (40,000 spermatozoa/mL) was measured using 2′,7′-dichlorodihydrofluorescein diacetate (H 2 DCF-DA) 2μM as previously described [30]. Within the cell, the esterase cleaves the acetate groups on H2DCF-DA, thus trapping the reduced form of the probe 2′,7′-dichlorodihydrofluorescein (H2DCF). Intracellular ROS oxidise H2DCF, yielding the fluorescent product, DCF. Following a 30 min incubation period at 37 °C, the fluorescence signal corresponding to DCF formation was recorded using a FLUO star Omega microplate reader (BMG LABTECH, Ortenberg, Germany). All fluorescence measurements (RFU = Relative Fluorescence Unit) were corrected for background fluorescence. Data were expressed RFU mean/number of spermatozoa ± SD [30].

##### Cell Extract and Total Thiols Level Assessment

After thawing, semen samples (200 µL 300 × 10^6^ total spermatozoa) were centrifuged at 1500× *g* for 10 min. The resulting pellets were treated for cellular extraction using PBS containing 0.1% Triton X-100 (500 μL). The extract was used for assaying total thiols as follows.

Total thiols were measured in cellular extracts using Ellman’s Reagent, 5,5-dithiol-bis-(2-nitrobenzoic acid) (DTNB) dissolved in PBS. Thiols were quantified spectrophotometrically using a Thermo Elecrom Corporation Genesys 10 UV spectrophotometer (Thermo Fisher Scientific, Rodano, Milano, Italy), at 412 nm, with an extinction coefficient of 14.150 M^−1^ cm^−1^. The values of Thiols in the samples were expressed in mmol/10^6^ spermatozoa [30].

##### Lipid Peroxidation

Lipid peroxidation was quantified by determining malondialdehyde (MDA) via the Thio barbituric acid reactive substances (TBARS) assay as previously described by Wirastuti et al. [33]. Following thawing, semen samples were centrifuged at 1600× *g* at 4 °C for 10 min then, 200 µL of cryopreservation medium were recovered and mixed with 200 µL of 20% trichloroacetic acid (TCA) solution containing 0.5% thiobarbituric acid (TBA). Next, 100 µL of hydrochloric acid 1N (HCl) and 500 µL of distilled water were added. The mixture was homogenized and incubated in a 95 °C water bath for 15 min to allow formation of the TBA-MDA complex. The samples were immediately cooled on ice and centrifuged at 10,000 rpm for 10 min at 4 °C. The resulting supernatant was collected, and absorbance was measured at 532 nm.

### 2.7. In Vitro Fertilization (IVF) Assay

The IVF assay was performed as previously described by Ebrahimi et al. [37]. Briefly, ovaries were collected from slaughtered ewes during the breeding season. Cumulus–oocyte complexes (COCs) were recovered by slicing antral follicles larger than 2 mm. Only oocytes with homogeneous cytoplasm and at least two compact layers of cumulus cells were selected for in vitro maturation (IVM).

Oocytes were matured in bicarbonate-buffered TCM-199 supplemented with 4 mg/mL bovine serum albumin (BSA), 100 µM cysteamine, 0.3 mM sodium pyruvate, 1 µg/mL estradiol-17β, and Pluset 1 IU/mL. Groups of approximately 30 oocytes were cultured per well for 24 h at 38.5 °C under 5% CO_2_ in a humidified atmosphere. After maturation, cumulus cells were removed by repeated gentle pipetting.

For IVF, frozen semen straws from the SP + 0, SP + 0.5, and SP + 2 groups were used. These groups were selected based on the results of the post-thaw membrane integrity and oxidative status analyses (ROS, MDA, and total thiols). Although seminal plasma removal did not significantly affect membrane integrity, SP− samples showed marked oxidative imbalance, characterized by increased ROS and MDA levels and reduced total thiol concentrations. Therefore, IVF was performed only with semen samples containing seminal plasma in order to preserve a more protective environment while evaluating the effect of the plant extract. Because oxidative stress is known to impair sperm fertilizing ability and early embryo development, IVF was intentionally performed only with SP+ samples to provide a physiologically relevant environment and to specifically evaluate whether *S. oleraceus* supplementation could further improve fertilization outcomes under conditions considered suitable for embryo production.

Semen straws were thawed at 37 °C for 30 s, then swim-up in Synthetic Oviductal Fluid (SOF) at 38.5 °C and 5% CO_2_ for 1 h. Oocytes were inseminated in 50 µL drops of SOF supplemented with 20% heat-inactivated estrous ewe serum at a final concentration of 1 × 10^6^ spermatozoa/mL (10 oocytes per droplet). Gametes were co-incubated for 24 h at 38.5 °C under 5% CO_2_ in a humidified atmosphere.

After fertilization, presumptive zygotes were washed and cultured in a four well dish with 500 μL culture medium (G-TL, Vitrolife, Gothenburg, Sweden) overlayed with 200 μL mineral oil. Embryos were cultured at 38.5 °C in a humidified atmosphere of 7% O_2_, 5% CO_2_, and 88% N_2_. Embryo development was assessed by calculating cleavage and blastocyst rates as follows: Cleavage rate = (Number of cleaved embryos/Total number of oocytes inseminated) × 100, Blastocyst rate = (Number of embryos reached the blastocyst stage/Total number of oocytes inseminated) × 100.

### 2.8. Statistical Analysis

A linear mixed-effects model was fitted to sperm motility and kinetics parameters, membrane integrity, biochemical and oxidative analyses (MDA, ROS, and total thiols) with presence or absence of seminal plasma, dose, all two-way interactions considered. Replicate (straws number) was included as a random intercept to account for non-independence among repeated observations. Model simplification was performed by removing non-significant interaction terms based on likelihood ratio tests. The reduced model did not differ significantly from the full model and was therefore retained.

Y_ijl_ = μ + SP_i_ + Dose_j_ + (SP × Dose)_ij_ + R_l_+ ε_ijl_

where

- μ is the overall mean;
- SP_i_ is the fixed effect of seminal plasma (SP−, SP+);
- Dose_j_ is the fixed effect of *Sonchus oleraceus* dose (0, 0.5, 2 g/L);
- (SP × Dose)_ij_ is the interaction between seminal plasma and dose;
- R_l_ is the random effect of replicate;
- ε_ijl_ is the residual error R_l_ is the random effect of replicate.

Model assumptions were assessed by visual inspection of residuals versus fitted values and normal quantile–quantile plots, indicating homoscedasticity and approximate normality of residuals. The random effect of replicate (R_l_) and the residual errors (ε_ijl_) were assumed to be normally distributed with mean zero and variances σR2 and σ2, respectively.

For the in vitro fertilization outcomes, only semen samples containing seminal plasma (SP+) were used. Therefore, seminal plasma presence/absence and the SP × dose interaction were not included in the statistical model for cleavage and blastocyst rates. A separate linear mixed-effects model was fitted to evaluate the effect of *Sonchus oleraceus* extract dose (0, 0.5, and 2 g/L) on cleavage and blastocyst rates. Three independent IVF replicates were performed.

Y_jl_ = μ + Dose_j_ + R_l_ + ε_jl_

where

- μ = overall mean;
- Dose_j_ = fixed effect of *Sonchus oleraceus* dose (0, 0.5, and 2 g/L);
- R_l_ = random effect of replicate;
- ε_j_ = residual error.

The significance of the fixed effects was assessed at a probability level of *p* < 0.05.

## 3. Results

### 3.1. Extract Yield and Total Polyphenol Content

The aqueous extraction yield of *Sonchus oleraceus* was 14.1 ± 0.6% and the total polyphenol concentration of the aqueous extract was about 193.4 ± 5.2 mg GAE/100 mL.

### 3.2. Post-Thaw Sperm Quality

#### 3.2.1. Effect of Seminal Plasma Removal on Sperm Kinematic Parameters

The presence of SP significantly modulated post-thaw sperm kinematic profiles (Table 1). Spermatozoa cryopreserved in the SP− samples exhibited significantly higher values of curvilinear velocity (VCL), amplitude of lateral head displacement (ALH), distance of the curvilinear trajectory (DCL), sperm head area, elongation and a lower percentage of abnormal morphology compared to the SP+ group (*p* < 0.05).

In contrast, SP+ samples showed significantly higher linearity (LIN), straightness (STR), and beat cross frequency (BCF). No significant differences were observed between the two groups regarding total motility, progressive motility, straight-line distance (DSL) and Straight-line Velocity (VSL).

#### 3.2.2. Impact of Seminal Plasma Removal on Oxidative Stress

**Figure 2:**
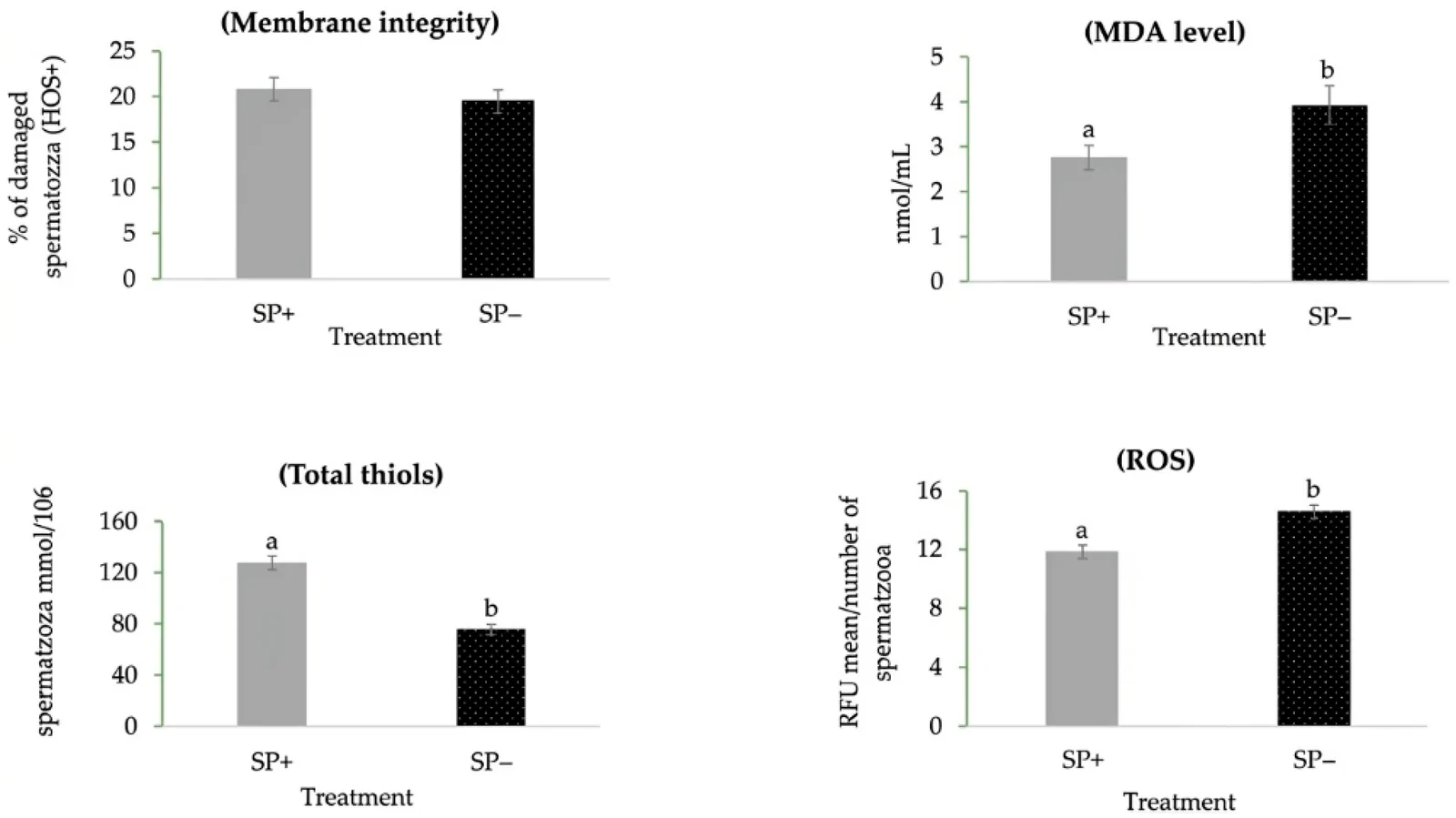
Effect of seminal plasma removal on membrane integrity, MDA level, total thiols and reactive oxygen species of post-thaw ram semen (SP+: presence of seminal plasma, SP−: absence of seminal plasma. Values are presented as means ± SEM for each group. Bars with different superscript letters (a,b) indicate a significant effect of seminal plasma presence or absence (*p* < 0.05).

SP removal did not affect post-thaw membrane integrity (*p* > 0.05); however, it markedly affected the oxidative status of post thaw spermatozoa. SP- samples showed significantly higher ROS levels, increased MDA concentrations and reduced total thiols content compared to SP+ samples (*p* < 0.05) (Figure 2).

#### 3.2.3. Effect of *Sonchus oleraceus* Aqueous Extract Supplementation on Sperm Kinematic Parameters

Supplementation of the semen extender with *Sonchus oleraceus* aqueous extract had negligible effects on most kinematic parameters. No significant alterations were observed in total motility, progressive motility, VCL and VSL between treatment groups. However, a deleterious effect was observed for the percentage of spermatozoa with abnormal morphology at the highest dose 2 g/L. Supplementation with 2 g/L of *S. oleraceus* extract significantly increased STR values in SP− samples (*p* < 0.05) (Table 2).

#### 3.2.4. Antioxidant Effect of *Sonchus oleraceus* Supplementation on Oxidative Stress Biomarkers

**Figure 3:**
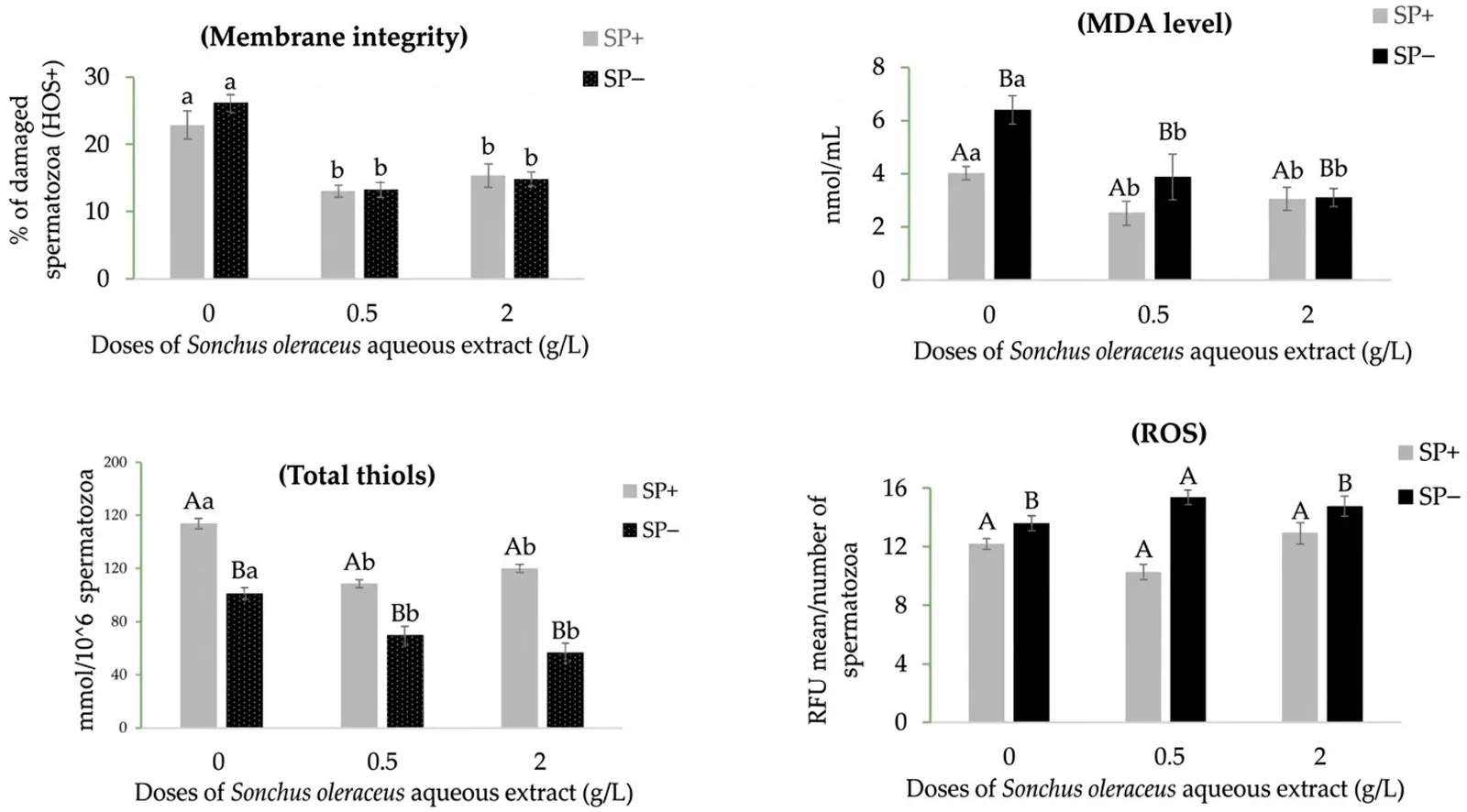
Effect of the incorporation of *Sonchus oleraceus* aqueous extract under seminal plasma presence and absence conditions on membrane integrity, MDA level, total thiols and reactive oxygen species of post-thaw ram semen (SP+: presence of seminal plasma, SP−: absence of seminal plasma. Values are means ± SEM in each group; bars with different superscript letters (A,B) differ significantly refer to the presence or absence of seminal plasma effect and different superscripts (a,b) differ significantly refer to the semen extender supplementation with *Sonchus oleraceus* aqueous extract effect (*p* < 0.05)).

Unlike its limited effects on sperm kinematics, *Sonchus oleraceus* supplementation of the semen extender, affected sperm membrane integrity, as indicated by a significant reduction in the percentage of damaged spermatozoa at 0.5 and 2 g/L compared with the control, with no significant difference between the two extract concentrations. Also, this supplementation modulated the oxidative status. Also, *Sonchus oleraceus* supplementation affected oxidative status. Total thiol concentrations differed among treatments, with no significant differences between the 0.5 and 2 g/L doses. MDA concentrations were lower at 0.5 and 2 g/L than in the control (*p* < 0.05), while no significant difference was detected between the two extract concentrations (Figure 3).

#### 3.2.5. Interaction Between Seminal Plasma Presence and *Sonchus oleraceus* Aqueous Extract Supplementation

No significant interaction between seminal plasma presence and *Sonchus oleraceus* extract supplementation was observed for sperm motility parameters.

However, significant interactions were found for ROS levels (*p* = 0.002) and MDA levels (*p* = 0.01). While SP− alone did not significantly affect membrane integrity, *Sonchus oleraceus* extract supplementation significantly affected membrane integrity at 0.5 and 2 g/L compared with the control (0 g/L; *p* = 0.02). No significant difference was detected between the two extract doses. Regarding oxidative status, MDA concentrations were lower at 0.5 and 2 g/L than in the control, whereas total thiol concentrations differed among treatments without a progressive dose-related response. In SP+ conditions, the responses to 0.5 and 2 g/L varied according to the parameter evaluated, with no significant difference between these doses for the oxidative stress parameters considered (Table 3).

### 3.3. Effect of Sonchus oleraceus Aqueous Extract Supplementation on In Vitro Embryo Development

Based on the results obtained for oxidative stress biomarkers and membrane integrity only SP+ samples were selected for in vitro fertilization assays. The high ROS and lipid peroxidation levels in SP− samples rendered them unsuitable for further developmental analysis.

**Figure 4:**
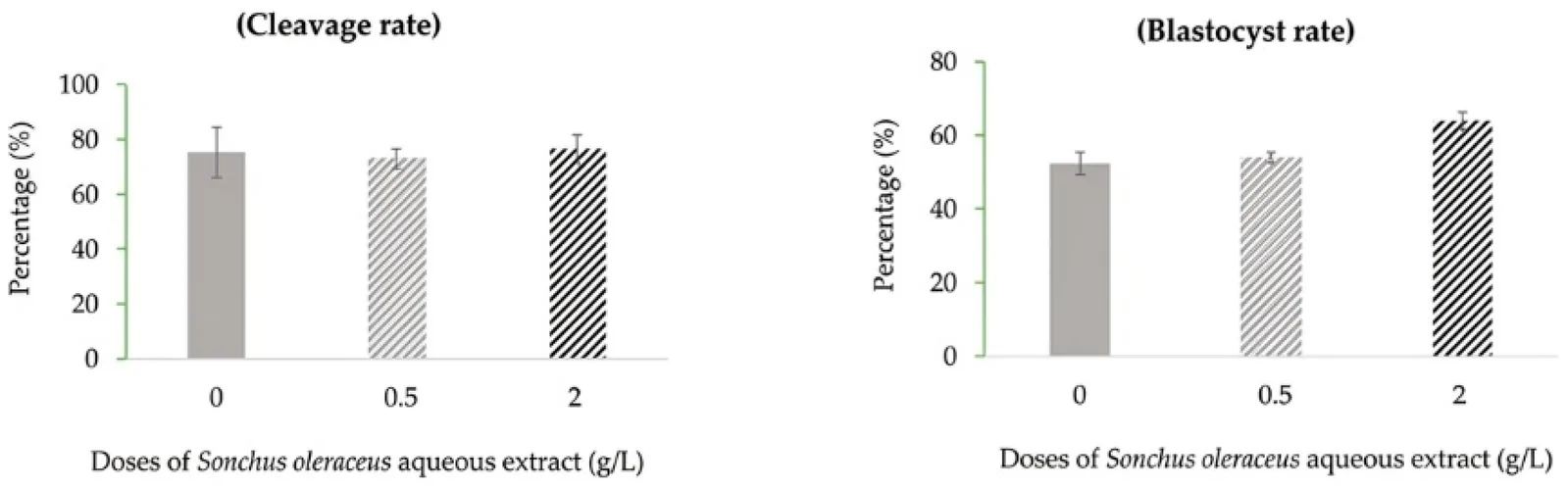
Effect of different concentrations of *Sonchus oleraceus* aqueous extract on in vitro embryonic development: Cleavage rate and blastocyst rate (Values are presented as means ± SEM for each group).

Cleavage rates were 75.43 ± 9.18%, 73.31 ± 3.45%, and 76.70 ± 5.04% respectively for control, 0.5 g/L, and 2 g/L *S. oleraceus* extract groups. Any significant effect of the extract supplementation was observed (*p* > 0.05). Blastocyst rates were 52.41 ± 2.87%, 54.18 ± 1.35%, and 63.77 ± 2.29% in the control, 0.5 g/L, and 2 g/L groups, respectively. No significant differences were detected in blastocyst rates among the experimental groups (*p* > 0.05), (Figure 4).

## 4. Discussion

The present study shows the complex and context-dependent role of SP in regulating sperm motility and redox homeostasis. The presence of SP significantly enhanced linear motility (LIN, STR) and beat cross frequency (BCF) (*p* < 0.05). Conversely, SP removal increased curvilinear velocity (VCL), amplitude of lateral head displacement (ALH), sperm head area, distal curvilinear length (DCL), and elongation, suggesting that spermatozoa express their intrinsic kinetic potential in the absence of SP. These changes may reflect, on the one hand, the important role of SP in maintaining coordinated and linear sperm motility patterns that are essential for fertilization [38]. These are likely mediated by specific proteins in SP that interact with the sperm membrane, stabilizing membrane structure and regulating fluidity [38,39], supporting an adaptive response to cryogenic stress, enhancing the ability to penetrate the zona pellucida [40,41]. However, excessive motility may indicate capacitation instability, as hyperactivation is associated with increased energy expenditure and membrane destabilization [42]. SP removal was associated with a significant reduction in abnormal morphology after freezing, indicating that SP removal allows spermatozoa to enhance certain morphological traits. This finding may reflect the complex composition of seminal plasma and its interaction with spermatozoa during cryopreservation, as some seminal plasma proteins can protect sperm structures, whereas their effects may vary depending on the freezing conditions and extender composition [8,12]. Ram sperm cells are highly susceptible to oxidative damage because their plasma membranes are rich in polyunsaturated fatty acids and characterized by a relatively low cholesterol-to-phospholipid ratio [18,43]. Removal of SP markedly increased reactive oxygen species (ROS) and lipid peroxidation (MDA) while reducing total thiols content (*p* < 0.05), highlighting the dependence of ram spermatozoa on extracellular antioxidant systems to preserve membrane integrity and functional competence during cryopreservation [16,44]. SP constitutes the primary extracellular antioxidant environment surrounding sperm, containing enzymatic antioxidants (superoxide dismutase, catalase, glutathione peroxidase) and non-enzymatic scavengers (glutathione, urate, ascorbate, taurine) that maintain redox balance during semen processing [45].

Supplementation with *Sonchus oleraceus* aqueous extract affected oxidative status, with significant differences observed for some oxidative stress parameters between the control and supplemented groups. The extract increased total thiols, decreased MDA, and preserved membrane integrity, indicating direct antioxidant activity, likely due to its polyphenol and flavonoid content, depending on the extraction solvent, plant origin and analytical methodology, total phenolic contents ranging until 195 mg GAE/g and flavonoid contents ranging from 7 to 75 mg QE/g have been reported [46,47], which neutralizes ROS, interrupts peroxidative chain reactions, and chelates pro-oxidant metals [47,48,49]. Polyphenol and flavonoid also enhance membrane fluidity and resistance to osmotic and thermal stress, maintaining post-thaw sperm functional competence [18,50].

A key finding of this study was the significant interaction between seminal plasma (SP) status and *S. oleraceus* extract dose for ROS, MDA, and HOS. This interaction indicates that the effect of extract supplementation depends on the presence or absence of SP. However, the present results do not support a dose-dependent or progressive protective effect, as the responses observed at 0.5 and 2 g/L were not consistently different. SP may provide an immediate extracellular antioxidant defense through its enzymatic and non-enzymatic antioxidant components, thereby limiting the accumulation of reactive oxygen species and protecting spermatozoa against oxidative damage during cryopreservation [51,52]. Simultaneously, plant polyphenols may enhance intracellular redox buffering capacity by scavenging free radicals, chelating pro-oxidant ions, and reducing lipid peroxidation [53]. The combination of both mechanisms optimizes sperm resistance to cryodamage. Interestingly, no significant SP × extract interaction was observed for the sperm velocity descriptors, despite the significant interactions detected for ROS, MDA, and membrane integrity. This finding suggests that, under the conditions of the present study, the antioxidant protection provided by *S. oleraceus* did not translate into measurable changes in post-thaw sperm kinematics. This may be related to the multifactorial regulation of sperm motility, which depends not only on redox homeostasis but also on membrane protein organization, mitochondrial ATP production, and signaling pathways involved in sperm activation and capacitation. Thus, reducing oxidative damage may preserve cellular integrity without necessarily being sufficient to restore motility after cryopreservation [9].

Based on the oxidative imbalance induced by SP removal, the in vitro fertilization assay was intentionally performed only in the presence of SP to maintain basal antioxidant protection and physiologically relevant motility patterns. *Sonchus oleraceus* aqueous extract supplementation did not significantly affect either cleavage or blastocyst rates. This finding contrasts with previous reports describing beneficial effects of other plant extracts like *Moringa oleifera* seed extract [42] and *Lavender ethanolic* extract [53]. These findings confirm that plant-derived antioxidants mitigate cryopreservation-induced oxidative stress and improve physiological parameters [54]. These effects are attributed to their antioxidant and anti-inflammatory properties, linked to the presence of phenolic compounds able to reduce lipid peroxidation and stabilize the sperm plasma membrane [18]. Therefore, the present results do not provide evidence of an effect of extract supplementation on early embryonic development [18,55,56]. Although some numerical differences were observed among the treatment groups, these differences should not be interpreted as evidence of an optimal intermediate extract dose unless they are supported by statistically significant pairwise comparisons. In the present study, the results therefore support an interaction between SP status and extract supplementation for some oxidative and membrane-related parameters, but they do not establish a specific dose as optimal. Maintaining physiological redox balance is crucial for preserving motility, membrane integrity, and fertilizing potential [53,54]. The absence of statistical significance may be related, at least in part, to the limited number of *IVF* replicates and/or oocytes available for this analysis. Further experiments using a larger number of biological replicates and oocytes will be necessary to determine whether this apparent increase represents a reproducible effect of *S. oleraceus* supplementation.

Collectively, these results demonstrate that SP and *Sonchus oleraceus* extract act through complementary pathways to enhance resistance to oxidative stress and preserve sperm membrane integrity. This integrative strategy provides a robust framework for cryopreservation protocols in ram spermatozoa, addressing their specific oxidative vulnerabilities.

Although the present study demonstrated beneficial effects of seminal plasma and aqueous extract of *Sonchus oleraceus* on post-thaw ram sperm quality, several limitations should be considered, such as the experimental design focused primarily on post-thaw sperm quality and in vitro fertilization parameters, while long-term embryo development and pregnancy outcomes were not investigated. Furthermore, the specific bioactive compounds of the plant extract responsible for the observed protective effects remain unidentified. Finally, the assessment was mainly limited to conventional sperm quality parameters and in vitro fertilization outcomes, without exploring fertility performance by in vivo artificial insemination trials.

## 5. Conclusions

The present study indicates that seminal plasma and *Sonchus oleraceus* aqueous extract have distinct and interactive effects on the quality of cryopreserved ram spermatozoa. Seminal plasma contributed to the maintenance of coordinated sperm motility patterns and extracellular antioxidant protection, whereas its removal was associated with increased oxidative stress despite differences in some kinematic parameters, while no significant differences were observed in total and progressive motility. Supplementation with *S. oleraceus* aqueous extract improved oxidative status and membrane integrity after cryopreservation. Significant SP × extract dose interactions were observed for selected oxidative stress and membrane integrity parameters, indicating that the effect of the extract depended, at least in part, on seminal plasma status.

In the *IVF* assay, which was conducted only with SP+ semen, cleavage rates were not significantly affected by extract supplementation. Therefore, these *IVF* findings should be considered preliminary and do not provide sufficient evidence to conclude that *S. oleraceus* improves embryo development or functional fertility. Further studies with a larger number of independent biological and *IVF* replicates are required to confirm these observations and to investigate the mechanisms underlying the interaction between seminal plasma and plant-derived antioxidants.

## Figures and Tables

**Table 1:** Effect of seminal plasma on ram semen motility and velocity parameters following freeze–thawing.

Parameters	SP+ Group	SP− Group	*p*-Value
Abnormal morphology	18.8 ^a^ ± 0.7	14.8 ^b^ ± 0.7	<0.001
TM	41 ± 1.2	42.4 ± 1.7	0.300
Prog	25.6 ± 0.7	24.8 ± 0.9	0.410
ALH	6.2 ^a^ ± 0.0	6.9 ^b^ ± 0.1	<0.001
AREA	15.5 ^a^ ± 0.1	15.9 ^b^ ± 0.2	0.004
BCF	36.4 ^a^ ± 0.2	35.2 ^b^ ± 0.4	0.010
DAP	42.9 ± 0.3	42.2 ± 0.5	0.120
DCL	63.8 ^a^ ± 0.6	66.7 ^b^ ± 0.8	0.007
DSL	40.5 ± 0.3	39.5 ± 0.5	0.130
Elongation	0.46 ^a^ ± 0.002	0.47 ^b^ ± 0.002	0.030
LIN	66.7 ^a^ ± 0.3	62.7 ^b^ ± 0.5	<0.001
STR	94.8 ^a^ ± 0.0	94.2 ^b^ ± 0.0	<0.001
VCL	167.6 ^a^ ± 1.3	176.6 ^b^ ± 2.3	<0.001
VSL	107.1 ± 0.8	105.7 ± 0.8	0.240

SP+: presence of seminal plasma, SP−: absence of seminal plasma, Abnormal morphology (%)—percentage of spermatozoa with abnormal morphology, TM (%)—total motility, Prog (%)—progressive motility, ALH (µm)—amplitude of lateral head displacement, Area (µm^2^)—sperm head area, BCF (Hz)—beat cross frequency, DAP (µm)—average path distance, DCL (µm)—curvilinear distance, DSL (µm)—straight-line distance, Elongation (%)—sperm head elongation, LIN (%)—linearity (VSL/VCL × 100), STR (%)—straightness (%), VCL (µm/s)—curvilinear velocity, VSL (µm/s)—straight-line velocity. Means with different superscripts (a,b) within the same row differ significantly referring to the presence or absence of seminal plasma effect (*p* < 0.05).

**Table 2:** Effects of *Sonchus oleraceus* aqueous extract supplementation on post-thaw sperm motility and velocity parameters according to seminal plasma presence or absence.

Parameters	Seminal Plasma Treatment	Doses of *Sonchus oleraceus* Aqueous Extract (g/L)		
		0	0.5	2
Abnormal morphology	SP+	17.1 ^Aa^ ± 0.8	18.5 ^Aa^ ± 1.7	18.5 ^Ab^ ± 1.0
	SP−	13.1 ^Ba^ ± 0.8	13.4 ^Ba^ ± 0.8	18.4 ^Bb^ ± 1.5
TM	SP+	38.9 ± 3.6	40.1 ± 7.9	39.2 ± 3.5
	SP−	41.8 ± 4.5	37.3 ± 6.9	41.6 ± 11
Prog	SP+	24.5 ± 2.8	24.9 ± 2.8	26.0 ± 4.0
	SP−	24 ± 0.8	22.7 ± 2.1	24.6 ± 4.6
ALH	SP+	6.1 ^A^ ± 0.5	6.4 ^A^ ± 0.7	6.2 ^A^ ± 0.7
	SP−	6.6 ^B^ ± 0.9	7.3 ^B^ ± 0.8	6.3 ^B^ ± 0.9
BCF	SP+	36 ^A^ ± 0.7	35.5 ^A^ ± 1.5	36.3 ^A^ ± 1.1
	SP−	35.9 ^B^ ± 2.1	34.1 ^B^ ± 1.5	36 ^B^ ± 1.9
DCL	SP+	65.5 ^A^ ± 2.9	64.2 ^A^ ± 3.7	64.8 ^A^ ± 2.9
	SP−	66.4 ^B^ ± 3.4	70.1 ^B^ ± 3.2	63.1 ^B^ ± 4.3
DSL	SP+	41.5 ^A^ ± 1	40.1 ^A^ ± 1.7	41.4 ^A^ ± 0.7
	SP−	39.7 ^B^± 3.5	41.3 ^B^ ± 4.8	38.0 ^B^± 0.9
LIN	SP+	66.7 ^A^ ± 2.3	66.0 ^A^ ± 2.6	67.1 ^A^ ± 3.2
	SP−	63.2 ^B^ ± 2.5	62.3 ^B^ ± 4	64.1 ^B^ ± 3.8
STR	SP+	94.8 ^Aa^ ± 0.2	94.6 ^Aa^ ± 0.6	94.8 ^Ab^ ± 0.5
	SP−	94.4 ^Ba^ ± 0.4	93.7 ^Ba^ ± 0.9	94.9 ^Bb^ ± 1.1
VCL	SP+	168.3 ^A^ ±5.3	168.5 ^A^ ± 9.6	172.5 ^A^ ± 7.5
	SP−	175.3 ^B^ ± 7.2	186.1 ^B^ ± 7.4	167.5 ^B^ ± 12
VSL	SP+	107.6 ± 3.8	106.9 ± 6.6	110.3 ± 2.2
	SP−	105.7 ± 1.2	110.4 ± 4.8	102.2 ± 0.9

Abnormal morphology (%)—percentage of spermatozoa with abnormal morphology; TM (%)—total motility; Prog (%)—progressive motility; ALH (µm)—amplitude of lateral head displacement; BCF (Hz)—beat cross frequency; DCL (µm)—curvilinear distance, DSL (µm)—straight-line distance; LIN (%)—linearity (VSL/VCL × 100); STR (%)—straightness (VSL/VAP × 100); VCL (µm/s)—curvilinear velocity; VSL (µm/s)—straight-line velocity. SP+: presence of seminal plasma; SP−: absence of seminal plasma. Values are estimated marginal means ± SEM. Significant differences were determined using Tukey-adjusted pairwise comparisons (*p* < 0.05). Different uppercase superscript letters (A,B) indicate significant differences according to the presence or absence of seminal plasma, whereas different lowercase superscript letters (a,b) indicate significant differences among extract doses (*p* < 0.05).

**Table 3:** Interactive effect of seminal plasma status and *Sonchus oleraceus* aqueous extract supplementation on oxidative stress markers and sperm membrane integrity after ram sperm cryopreservation.

Parameters	SP (*p*-Value)	Dose (*p*-Value)	SP × Dose Interaction (*p*-Value)
Membrane integrity	0.330	<0.001	0.020
MDA	0.0003	<0.001	0.010
Total thiols	<0.001	0.0001	0.210
ROS	<0.001	0.130	0.002

SP (Seminal plasma; SP+: seminal plasma present and SP−: seminal plasma removed); MDA: Malondialdehyde level; ROS: reactive oxygen species level.

## Data Availability

The data presented in this study are available on request from the corresponding author.

## Abbreviations

The following abbreviations are used in this manuscript:

Abbreviation	Full term
SP	Seminal Plasma
CASA	Computer-Assisted Sperm Analysis
MDA	Malondialdehyde
ROS	Reactive Oxygen Species
HOS	Hypo-Osmotic Swelling
IVF	In Vitro *Fertilization*
TM	Total motility
Prog	Progressive motility
ALH	Amplitude of lateral head displacement
Area	Sperm head area
BCF	Beat cross frequency
DAP	Average path distance
DCL	Curvilinear distance
DSL	Straight-line distance
LIN	Linearity (VSL/VCL × 100)
STR	Straightness (VSL/VAP × 100)
VAP	Average path velocity
VCL	Curvilinear velocity
VSL	Straight-line velocity
